# Development of multiplex serological assays to detect oncoviral infections

**DOI:** 10.1186/1750-9378-7-S1-P30

**Published:** 2012-04-19

**Authors:** William E Burgan, Katie Beam, Matthew Bess, Allison Meade, Katie Wakeham, Robert Newton, Denise Whitby, Rachel K Bagni

**Affiliations:** 1 Protein Expression Laboratory, Advanced Technology Program, SAIC-Frederick, Frederick, MD, USA; 2 Medical Research Council/Uganda Virus Research Institute Uganda Research Unit on AIDS, Entebbe, Uganda; 3 Epidemiology and Genetics Unit, Department of Health Sciences, University of York, York, UK; 4 Viral Oncology Section, AIDS and Cancer Virus Program, SAIC-Frederick, Frederick, MD, USA

## 

Serological markers of infection (antibodies or antigens) of viruses that cause cancer are most often detected using ELISA-based methodologies. In many cases, multiple markers of infection must be assessed to determine a final sero-status. Volume requirements and costs of reagents for single analyte ELISAs are high and studies which include multiple viruses can require milliliters of plasma, often not available from archived cohorts. Thus, we sought to develop a Luminex^®^ bead-based customizable panel initially including Hepatitis B Virus (HBV), Hepatitis C Virus (HCV), Epstein-Barr Virus (EBV) and often the concomitant infection, Human Immunodeficiency Virus (HIV) to reduce sample volume requirements, overall cost and increase flexibility. Peptides, antigens and antibodies were sourced from multiple manufacturers and tested for suitability in this platform. Where necessary, suitable reagents were designed and produced in-house. The HCV assay multiplexes four HCV peptides designed to detect antibodies raised to HCV Core (2), HCV NS4, HCV NS5 gene regions. The HBV assay multiplexes a HBV early antigen peptide, a recombinant HBV core protein and either a recombinant HBV surface antigen or an antibody specific for HBV surface antigen to assess HBV infection. The EBV assay multiplexes peptides specific to viral capsid antigen, EBNA-1 and early antigen (Cyto-Barr, Zuidhorn, The Netherlands). HIV-1 assay development is ongoing and the list of antigens to be included in the assay has not been finalized. These assays can be run singly or with any combination (multi-plex) of the above listed targets. Each target has been independently validated using samples of known molecular and serological status to determine specificity (false positive versus false negative) and re-evaluated under multiplex conditions to confirm assay performance. In addition, where possible, samples were assayed on commercial testing platforms as well as our multiplex assay to assess concordance (94-99%). Dependent on the panel selected and the expected antibody titers in a particular population, plasma or serum volumes in the range of 10 µL to 125 µL per subject would be required to determine the HBV, HCV, EBV and/or HIV serostatus of a subject. This assay platform is inherently flexible and the benefits include amenability to expansion to include other oncogenic viruses as well as screening large epidemiological cohorts or smaller subsets of samples in an economical and high throughput manner.

